# Prior Expectation Modulates Repetition Suppression without Perceptual Awareness

**DOI:** 10.1038/s41598-018-23467-3

**Published:** 2018-03-22

**Authors:** Leonardo S. Barbosa, Sid Kouider

**Affiliations:** 1 Brain and Consciousness Group (ENS, EHESS, CNRS), École Normale Supérieure - PSL Research University, Paris, France; 2 École Doctorale Cerveau Cognition Comportement, Université Pierre et Marie Curie, Paris, France

## Abstract

Stimulus repetition induces attenuated brain responses. This phenomenon, termed repetition suppression (RS), is classically held to stem from bottom-up neuronal adaptation. However, recent studies suggest that RS is driven by top-down predictive mechanisms. It remains controversial whether these top-down mechanisms of RS rely on conscious strategies, or if they represent a more fundamental aspect of perception, coding for physical properties of the repeated feature. The presence of top-down effects in the absence of perceptual awareness would indicate that conscious strategies are not sufficient to explain top-down mechanisms of RS. We combined an unconscious priming paradigm with EEG recordings and tested whether RS can be modulated by the probability of encountering a repetition, even in the absence of awareness. Our results show that both behavioural priming and RS near occipital areas are modulated by repetition probability, regardless of prime awareness. This contradicts previous findings that have argued that RS modulation is a by-product of conscious strategies. In contrast, we found that the increase in theta-band power following unrepeated trials – an index of conflict detection – is modulated only by expectations during conscious primes, implicating the use of conscious strategies. Together, our results suggest that the influence of predictions on RS can be either automatic in sensory brain regions or dependent on conscious strategies.

## Introduction

Neural activity induced by a stimulus is usually reduced when it is repeated. This phenomenon, termed repetition suppression (RS), is classically attributed to neuronal adaptation^1,2^. RS has been traditionally assumed to result from a bottom-up modulation of neuronal sensitivity to the stimulus, either as a consequence of neuronal fatigue or through the sharpening of neural representations. For this reason, RS has been extensively used to investigate neuronal selectivity across a variety of recording modalities^1,3^. Simply put, the neurons showing a decrease in sensitivity following the repetition of a specific feature (e.g. shape, orientation, etc.) are assumed to code for this feature^4,5^.

However, RS has recently been argued to derive from top-down predictive mechanisms. From this perspective, perception emerges as a comparison between top-down activity (reflecting the expected percept) and bottom-up activity (resulting from the observed sensory evidence). The overall activity elicited by a stimulus would reflect the difference between the expected and the actual stimulus^6,7^. More specifically, the decrease in activity after the second presentation of a stimulus would be a consequences of a reduced ‘prediction error’ because this stimulus was previously seen and hence predicted (i.e., more probable), in comparison to a novel one^8,9^. This predictive coding framework makes the important additional assumption that RS should increase with the likelihood of encountering a repetition. This repetition probability effect (RPE) has been shown not only for faces^9,10^ but also for auditory tones^11^, suggesting that predictions play a crucial role in RS^12^.

Yet, this interpretation has been challenged by a surge of recent studies showing RS unaffected by repetition likelihood. For instance, while the RPE has been consistently reported for faces^9,10,13,14^, it hardly generalizes to everyday objects, natural scenes and meaningless shapes such as fractals^15,16^. Moreover, unlike RS, the RPE appears to vanish when the repeated stimulus becomes task-irrelevant^17^. The lack of RPE while RS is maintained, casts doubts on predictive coding accounts of RS and suggest that the RPE results from the manifestation of explicit and conscious strategies^17^.

However, it remains possible that the RPE requires conscious strategies only for building predictions and not for their use during online processing. Indeed, the predictive mechanisms underlying the RPE might require robust experience-dependant plasticity^18^, which is not attained for every perceptual conditions. On the other hand, after predictions have become stable, these predictive mechanisms might become pervasive and independent of conscious strategies. This hypothesis predicts that an RPE can be observed even under situations where the use of conscious strategies is impossible.

To address this issue, we combined EEG recordings with a repetition priming paradigm under conscious and unconscious perceptual conditions. We also varied the probability of repetitions between experimental blocks to examine how the neural signatures of repetition priming are modulated by predictions, even the absence of awareness. Although the link between the general decrease in activity following the repetition of a prime stimulus (neural priming) and the RS effect seen in fMRI studies is still under debate^2,19,20^, there is a strong correlation between neural priming and RS effects^20–23^. More specifically, while late effects in the frontal regions are more sensitive to stimulus-response associations, early occipitoparietal regions reflect more perceptual processing independent of stimulus-response^20,23^. Importantly, previous studies using event-related potentials (ERPs) have established that the neural signature of unconscious behavioural priming is a good proxy for RS, as it is associated with a decrease in amplitude in occipitoparietal electrodes after the repetition of a stimulus even when the prime is rendered invisible^24,25^.

Our results show that RS in earlier sensory regions is modulated by the likelihood of encountering a repetition, even in the absence of awareness. On the other hand, theta-band increase in medial frontal regions – a known marker of conflict^26–29^ – was present and modulated by predictions only during conscious trials. We also show that when participants are never presented with visible repetition trials, unconscious priming is maintained but not modulated by probabilistic contexts. These results argue for a dissociation between RPEs reflecting unconscious, automatized predictive processes, and those reflecting conscious strategies.

## Results

**Figure 1:**
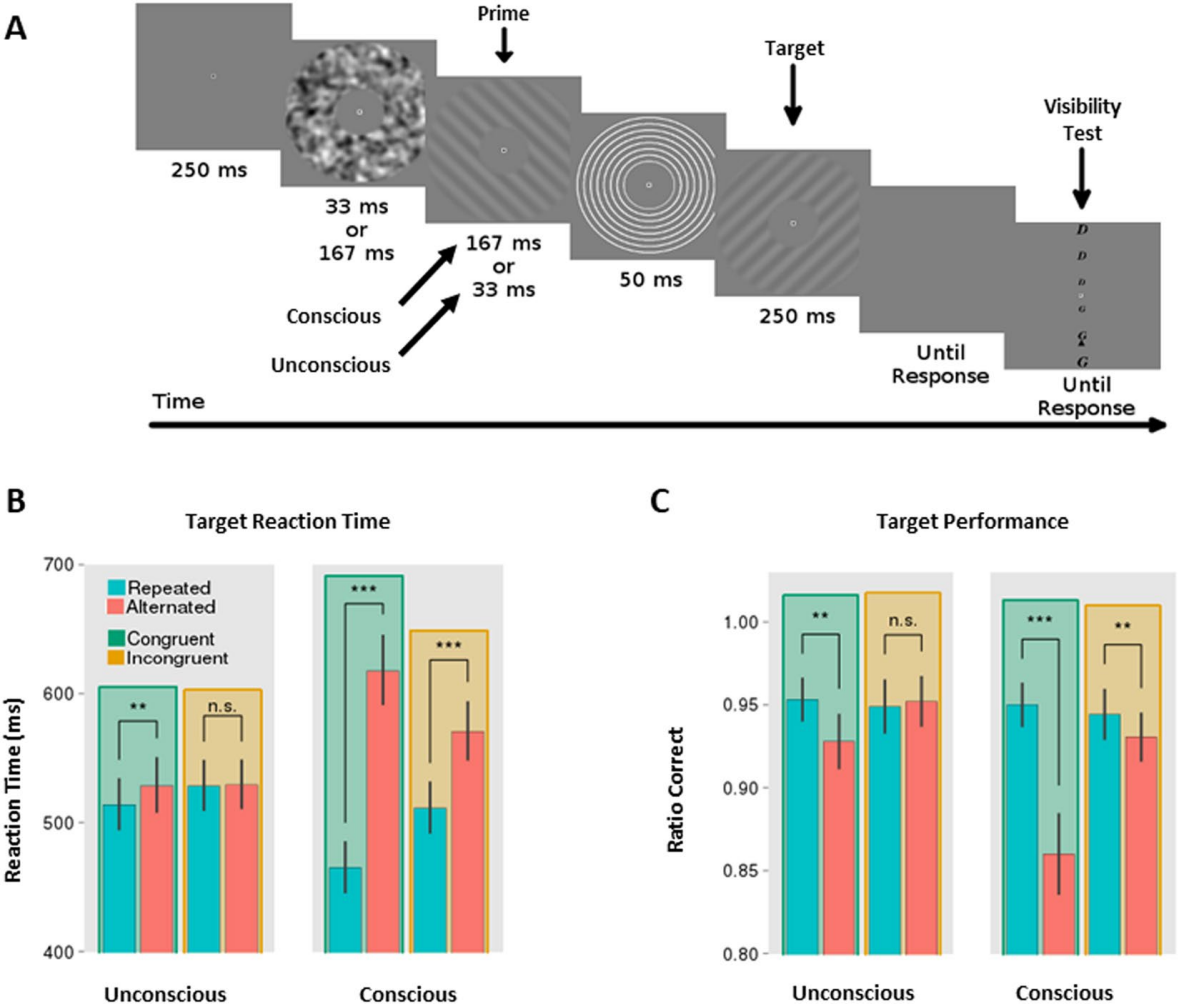
Paradigm and Priming. (**A**) In each trial, participants were presented with a sequence of two gratings inside an annulus, separated by a brief pattern mask composed of concentric circles. Each grating was either tilted to the left or to the right (−45 and +45 degrees, respectively). Participants were instructed to identify as fast as possible the direction of the second grating (the target). Following their response, they had to select as accurately as possible the direction of the first grating (the prime). Trials were either repeated (same orientation for prime and target) or unrepeated (opposite prime and target orientations), and were grouped in two types of blocs: 80% of repeated trials (congruent context) and 80% of unrepeated trials (incongruent context). Also, in each trial, the prime was either made invisible by presenting for 33 ms (unconscious condition) or visible by presenting for 167 ms (conscious condition). Figures (**B** and **C**) show reaction times and accuracy for the target discrimination task, as a function of prime visibility and probabilistic context (paired two-tailed t-tests, ns: p > 0.05, *p < 0.05, **p < 0.01, ***p < 0.001; error bars represent s.e.m.).

In order to investigate the effect of expectations on conscious and unconscious RS, we performed a series of 3 experiments. In each experiment, trials were composed by a sequence of gratings. The first grating was the prime and the second was the target on which participants performed a speeded orientation discrimination task (i.e., tilted to the left vs. right). The first two experiments had a 2 × 2 × 2 design: repetition (repeated vs alternated), context (congruent vs incongruent) and prime visibility (conscious vs unconscious; Fig. 1A) and were merged for behavioural analyses. During half the trials, the prime and target were the same stimulus (repeated condition) and during the other half they were composed of different stimuli (alternated condition). Trials occurred in blocks with a high 80% probability of repetition (congruent context) or a low 20% probability of repetition (incongruent context). In half the trials, the primes were heavily masked to render them unconscious while they were conscious during the other half of the trials. EEG was acquired during the second experiment. A third experiment was performed to investigate the impact of expectations on unconscious trials when participants are not consciously exposed to the probabilistic context through visible primes. For this reason, this third experiment had a 2 × 2 repetition (repeated vs alternated) x context (congruent vs incongruent) design and contained only unconscious priming trials. Unless stated otherwise, all statistical tests use an alpha level of 0.05, all t-tests are paired and two-tailed and all ANOVA analysis have within subject contrasts. Please refer to the Methods section for more details about the design and statistical analyses.

### Behavioural Results: Conscious and unconscious RPE

Participants were close to ceiling discriminating the target gratings [mean performance = 0.95; T(43) = 69, p = 1e-16], and performed fairly well discriminating the primes in the conscious condition [mean performance = 0.74; T(43) = 10, p = 1e-13]. However, as intended, they were at chance identifying the primes in the unconscious condition [mean performance = 0.50; T(43) = 1.03, p = 0.30]. A global ANOVA on reaction times (RTs) with prime visibility (conscious and unconscious primes), context (congruent and incongruent blocks) and repetition (repeated and alternated trials) as main factors revealed a significant three-way interaction [F(1, 38) = 37.32; p = 4e-7]. Separate analyses for each prime visibility level revealed a significant interaction between context and repetition for both conscious [F(1, 38) = 50.17; p = 1e-7] and unconscious [F(1, 38) = 6.72; p = 0.013] conditions. Further post-hoc analyses revealed conscious priming effects (faster RTs for repeated compared to unrepeated trials) for the congruent [T(38) = 8.68, p = 1e-10] and incongruent [T(38) = 5.97, p = 1e-06] conditions. Interestingly, they showed unconscious priming only during the congruent condition [T(38) = 2.96, p = 0.0052], but failed to reach significance during the incongruent context [T(38) = 0.22, p = 0.82; see Fig. 1B].

Similar analyses for accuracy rates also revealed a significant three-way interaction between prime visibility, context and repetition [F(1, 43) = 5.737; p = 0.021]. As with reaction times, separate analyses for each prime visibility level revealed a significant interaction between context and repetition for both conscious [F(1, 43) = 13.19; p = 0.00074] and unconscious [F(1, 43) = 7.01; p = 0.011] conditions. Priming effects (fewer errors for repeated compared to unrepeated trials) were significant for the conscious congruent [T(43) = −4.31; p = 9.2e-5], conscious incongruent [T(43) = −2.31; p = 0.025] and unconscious congruent [T(43) = −3.13; p = 0.0031] conditions, but not for unconscious primes in the incongruent context [T (43) = 0.37, p = 0.71; see Fig. 1C].

### EEG: Event-Related Potentials

**Figure 2:**
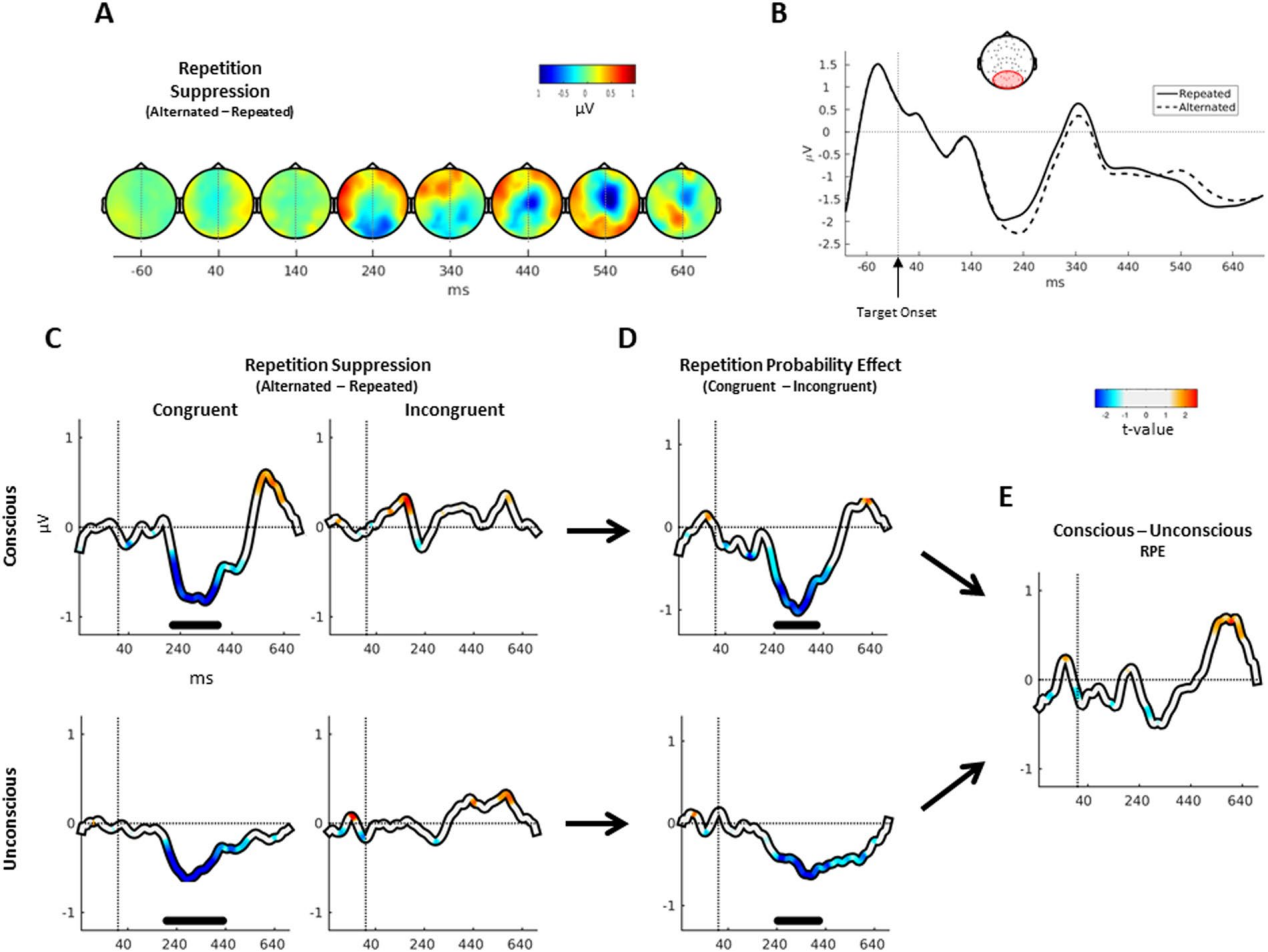
Evoked Potentials. (**A**) Scalp topographies showing the difference between ERPs (time-locked to the target onset) evoked by unrepeated and repeated trials (i.e. repetition suppression or RS) collapsed across context and visibility. The first component is observed around 240 ms over electrodes in the vicinity of occipital areas. (**B**) Time-plot showing the ERPs for repeated and unrepeated trials for these electrodes (collapsed across context and visibility). (**C**) RS effect for each prime visibility and context (i.e. difference between unrepeated and repeated ERPs in the selected electrodes): significant clusters found only during congruent context, peaking around 280 ms, for both levels of awareness. (**D**) Difference between RS for each context (or Repetition Probability Effect, RPE), for each level of prime visibility. Significant clusters were found regardless of prime awareness. (**E**) Difference between conscious and unconscious RPEs: no significant cluster was found. Time plots are smoothed with a 50 ms moving average window for visualization (only μV information have been smoothed, not t-values). Horizontal solid lines in time plots close to time axis indicate significant cluster (monte-carlo p-value < 0.05).

To avoid circular inference^30^, we selected electrodes showing a global RS effect independently from the factors of interest (i.e., prime visibility and context). As can be seen in Fig. 2A, a repetition suppression effect time-locked to the target onset was present over electrodes in the vicinity of occipital areas, starting around 240 ms (closest equivalents in the 10–20 system: O1, O2, Oz, PO3, PO4, PO7, PO8, POz, P3, P4, P5, P6, P7, P8). Figure 2B shows the time-plot ERPs for the repeated and unrepeated conditions over these electrodes.

We then inspected the three-way interaction between prime visibility, context and repetition using a cluster-permutation algorithm over the average activity of these electrodes. To this end, we first compute the RS effect during each context and visibility levels, i.e. the difference between alternated and repeated ERPs averaged across the electrodes of interest. We further compute the RPE effect by taking the difference between the RS effect during each visibility level. Finally, we calculated the pared t-statistics between the RPE effect during each visibility level (i.e. conscious and unconscious trials), and performed a cluster-permutation analysis to assess the significance of the effect (see Materials and Methods).

This analysis failed to show any significant cluster (Fig. 2E). However, when restricting our analysis to RPE effect (the difference between RS effect during congruent and incongruent trials) for each visibility level, we found significant clusters starting around 240 ms and ending around 440 ms both for unconscious (monte-carlo p-value = 0.0010, alpha-value = 0.025) and conscious primes (monte-carlo p-value = 0.0140, alpha-value = 0.025; Fig. 2D). Post-hoc analyses revealed that the RS effect (difference between alternated and repeated trials) in the congruent context for both unconscious (monte-carlo p-value = 0.0040) and conscious primes (monte-carlo p-value = 0.0090; Fig. 2C), peaking around 280 ms and evidencing higher amplitude for alternated trials (i.e. repetition suppression). However, there was no significant cluster when analysing the RS effect for unconscious or conscious primes during the incongruent context. This result demonstrates that RS can be modulated by the probability context independently of perceptual awareness.

### EEG: Time-Frequency

While behavioural priming can be described in terms of the perceptual mechanisms underlying RS, it can also reflect conflict detection when the prime and target are unrepeated, as they reflect alternating decisions engaging the cognitive control system. A hallmark of conflict detection and cognitive control is theta-band (i.e., 3–8 Hz) activity over medial-frontal electrodes^28,29,31–33^. In order to inspect whether conflict detection after unrepeated trials is modulated by prime visibility and context, we focused our EEG analyses on theta-band activity.

**Figure 3:**
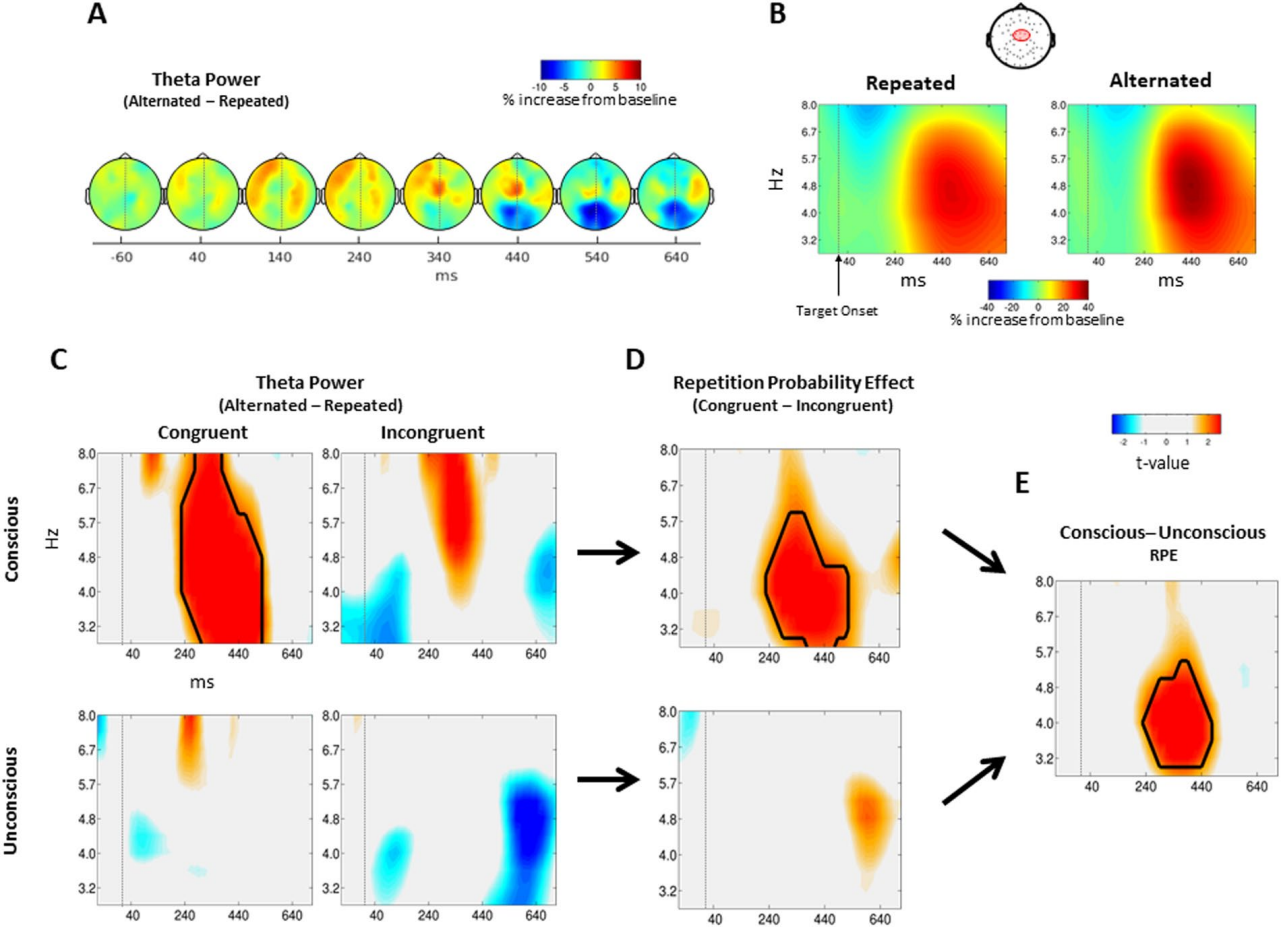
Theta Activity. (**A**) Scalp topographies showing the effect of repetition (unrepeated minus repeated trials) for Theta band activity (3–8 Hz) elicited at target onset, collapsed across context and visibility. (**B**) Time-Frequency plots showing the activity in medial-frontal electrodes. (**C**) Difference between unrepeated and repeated trials for each prime visibility and context (i.e. theta band increase) showing a significant cluster found only for conscious primes in the congruent context. (**D**) Differences between theta band increase for each context (or theta-band repetition probability effect, RPE) for each prime visibility. Significant cluster peaking around 4.5 Hz and 360 ms was revealed only during conscious condition. (**E**) Difference between conscious and unconscious RPEs, with a significant cluster peaking around 4.5 Hz and 360 ms. In all Time-Frequency figures, contours indicate significant clusters (monte-carlo p-value < 0.05).

A global analysis on power activity elicited after target onset confirmed that theta-band activity over medial-frontal sites was increased for unrepeated compared to repeated targets. This effect seems to occur later than RS in visual electrodes, starting around 340 ms (Fig. 3A). Again, this analysis allowed us to select the electrodes of interest in order to further explore the interaction between repetition, visibility and context (closest equivalents in the 10–20 system: FC1, FC2, FCz and Cz). Figure 3B shows the average power activity for repeated and unrepeated trials over these electrodes.

Similar to the ERP analyses, we first computed the theta-band power increase by taking the difference between alternated and repeat trials during each context and visibility levels. We then took the difference between the theta-band increase during congruent and incongruent contexts for each visibility levels, computing RPE effects for conscious and unconscious trials. Finally, we calculated the pared t-statistics between the theta-band RPE effect during each visibility level (i.e. conscious and unconscious trials), and performed a cluster-permutation analysis to assess the significance of the effect (see Materials and Methods).

This analysis revealed a significant interaction between prime visibility, context and repetition (monte-carlo p-value = 0.039) peaking around 4.5 Hz and ranging from 280 ms to 530 ms (Fig. 3E), which matches the known pattern of activity for conflict-related theta-band activity^33^. Further analyses revealed that this effect is solely driven by conscious priming (Fig. 3D), showing a significant cluster in the difference between congruent and incongruent contexts when the participants were aware of the prime (monte-carlo p-value = 0.017), but no significant cluster for the unconscious condition. More specifically, the theta-band increase was only significant during the congruent context for conscious primes (monte-carlo p-value = 0.0097), peaking around 360 ms, and was absent for all other conditions (Fig. 3C). This result shows that theta-band activity in medial-frontal sites, which is usually associated with conflict and learning^34,35^, is modulated by repetition only after a conscious prime. This finding suggests that conscious perceptual processing of the primes is necessary for updating or for sustaining the context after detecting a conflict^36–38^.

### Behavioural Results: Unconscious priming without intermixed conscious repetitions

In our previous experiments, both conscious and unconscious primes were intermixed in a single session. It therefore remains unclear whether conscious perceptual processing was necessary to build up the predictions underlying the RPE. In order to determine whether subjects had to be aware of the prime-target relationship to show an RPE, we ran a further experiment where subjects never saw the prime consciously. Subjects received exactly the same trial structure as in the previous experiment, except the primes were always unconscious to participants (i.e., presented for only 33 ms).

**Figure 4:**
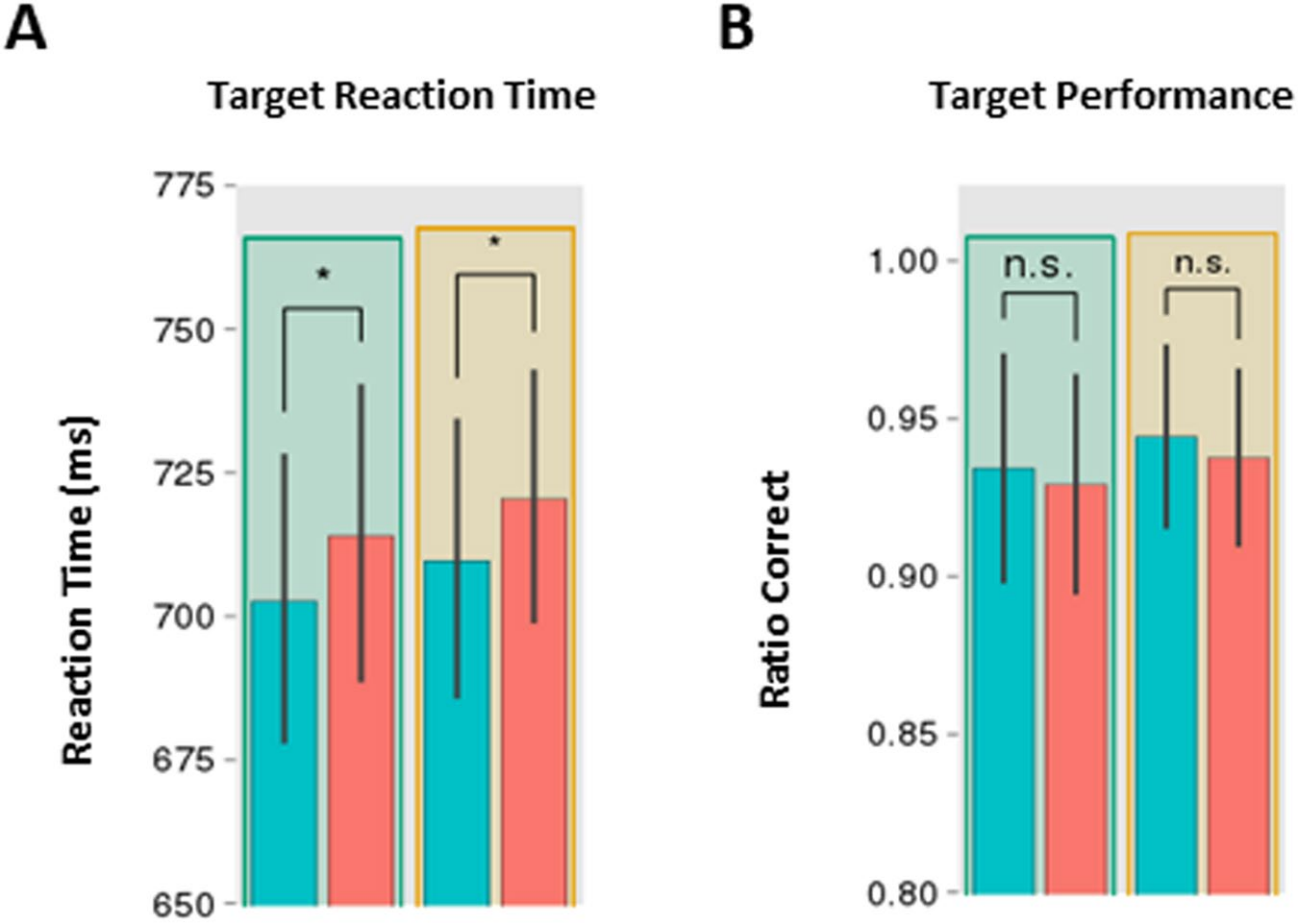
Unconscious priming without intermixed conscious repetitions. (**A**) shows reaction times and (**B**) shows accuracy for the target discrimination task, as a function of probabilistic context (paired two-tailed t-tests, ns: p > 0.05, *p < 0.05, **p < 0.01, ***p < 0.001; error bars represent s.e.m.).

Participants were again close to ceiling discriminating the target gratings [mean performance = 0.94; T(19) = 13.62, p = 2.9e-11], and also at chance identifying the primes [mean performance = 0.51; T(19) = 1.85, p = 0.079]. We next tested for an interaction between context and repetition. An ANOVA on reaction times (RTs) with factors as prime visibility and context failed to reveal a significant interaction between context and repetition [F(1, 19) = 0.013, p = 0.91] (Fig. 4A). Nevertheless, a main effect of priming was observed [F(1, 19) = 12.7; p = 0.0020]. Interestingly, contrary to the previous experiments, post-hoc analyses found priming not only for the congruent context [T(19) = 2.75, p = 0.012], but also for the incongruent context and [T(19) = 2.61, p = 0.017]. This result shows that priming was present independently of context, and suggests that consciousness is necessary to modulate priming based on the statistics of the environment (i.e. update of priors). Similar analyses on accuracy rates failed to show any significant effect or interaction (all p’s > 0.1; Fig. 4B).

## Discussion

Previous studies have shown that RS increases with the probability of encountering a repetition (i.e., the RPE). While this has been used as evidence that RS is a by-product of top-down predictions^9^, others have contested this assumption and argued the RPE is a by-product of conscious strategies^17^. Our EEG data reveal that the RPE can be observed even when subjects were not consciously aware of repetitions, suggesting that the involvement of conscious strategies is not sufficient to account for this phenomenon.

In the current study, the earliest RPE effect started around 240 ms and peaked around 280 ms. In the EEG modality, RS is often observed in occipitoparietal electrodes between 200 and 350 ms^23,39,40^. This component broadly overlaps with the posterior N2 component, which has also been shown to decrease after stimulus repetition^41^. Some studies have shown that additional repetitions of the target^42^, as well as attentional manipulations^43^, do not seem to further modulate the N2 component. For this reason, it has been argued that the N2 component reflects a first obligatory processing stage^42,43^. Interestingly, another studies have shown that RS in occipitoparietal components around 250 ms are sensitive to task relevance^23^ and stimulus familiarity^25,39,44^. Our results provide further evidence that RS in early occipitoparietal components are sensitive to cognitive manipulations, specifically to the probabilistic context in which the stimuli appear, even in the absence of perceptual awareness. Another possibility is that masking techniques may result in delayed ERP components^25^. Indeed, repetition of unfamiliar stimulus such as gratings^45^ and tones^11^ seem to elicit a reduction of activity for repeated stimulus already at 100 ms. However, in our study the RS effect did not occur until 200 ms. Due to the nature of the masking technique used in our manipulations, further studies employing other techniques such as Continuous Flash Suppression (CFS)^46,47^ may be necessary to disentangle these two hypotheses.

Importantly, here this early occipitoparietal ERP component was modulated independently of perceptual awareness, and therefore is independent of conscious strategies. Conversely, later theta-band activity, which is known to increase after a conflict^26–29^, was also modulated by the probability of repetitions, which is similar to previous results^10,11^. Crucially, not only our results show that theta band increase peaks later around 360 ms, but also that the modulation of this conflict effect occurs only in the conscious condition. This reveals an interesting dissociation between the early ERP component showing an RPE, and the later theta-band RPE. Furthermore, this is compatible with several studies showing that ACC is activated for conscious, but not for unconscious conflict^48–50^. Similarly, another study failed to observe changes in theta-band power related to unconscious conflict, even though they observed a change in connectivity between medial frontal and occipital sites in the theta-band^51^. It is important to mention that a recent study^52^ found that conflict related theta-band activity was modulated by previous trials’ conflicts (i.e. Gratton effect), even when the primes in the current and previous trials were unconscious. Even though further research will be necessary to understand how unconscious cognitive control relates to theta-band activity, it is clear that the earlier ERP effect and the later theta-band effect are contributing to different aspects of the RPE. While the later effects are more likely related to conscious strategies, the earlier effects are more likely related to the encoding of stimulus features. Those two aspects of RPE should be carefully considered in future research.

The theta-band activity in medial frontal sites has also been linked to learning and memory^34,35,53^. Consequently, this activity might be related to the updating of priors, which in turn would drive the automatization of predictive mechanisms, and result in the unconscious RPE effect we observed. Indeed, in a control experiment where only unconscious primes were present, we failed to observe modulation of repetition priming by the predictive context. This indicates that the presence of conscious primes during the experiment might be necessary to observe the probability effect, at least at the behavioural level. Previous studies reported modulation of unconscious priming by probabilistic contexts^36,54,55^. Interestingly, conscious conflicts are apparently necessary to drive this effect^55^ (but see ref.^56^). Finally, it is important to notice that during the control experiment, repetition priming was present in *both* contexts. This also argues against a lack of statistical power resulting in the absence of the RPE during the control experiment, since when the RPE is significant there is no significant unconscious priming during the incongruent context, although the sample size of the first two experiments combined is larger (see Supplementary Information).

We argue that a key aspect of the RPE is the joint build-up of stable predictive codes leading to robust prior expectations^18^ and involvement of higher-level mechanisms generally associated with conscious strategies^17^. Our results provide further support to the interpretation of RPE as reflecting a sequence of effects that might mingle and interact distinctively depending on the conditions^57^. For instance, a recent study using fMRI failed to replicate RPE at face-sensitive cortical regions, while revealing both modulation of activity in mid-prefrontal cortex and behavioural RPE^58^. In particular, our data suggests that the RPE reflect distinct involvements of lower-level perceptual processes and higher-level conscious strategies. A possible explanation as to why RS can sometimes be observed in the absence of RPE, is that both effects can be evoked independently and interact differently depending on the constraints of the protocol^57^. While our results are in line with a growing number of studies accounting for RS as the result of a mismatch between predictions and sensory evidence^9,12,59,60^, they also conflict with studies showing RS independently of predictive context^17,18^. Interestingly, while some of these studies did not observe RPEs for categories other than faces^15,61^, here we show that RPEs can be observed even for unfamiliar stimulus (i.e. Gabor patches). Arguably, this generalisation reflects thorough experience with this type of stimulus during the experiment^18^.

We propose that RS for a given class of stimuli is at first independent of probabilistic context, either through bottom-up adaptation^1^ or through the involvement of highly stable priors (e.g., capitalising on the general stability of the world)^62^. Later in time, following the entrainment of higher-level predictive mechanisms contingent on statistical regularities in the environment, RS might be modulated or even suppressed. We argue that higher-level predictive mechanisms would at first rely on conscious strategies. However, after extensive learning, even high-level predictions can be automatized and instantiated regardless of consciousness. This automatization would take place at different stages in the hierarchy of processing levels^7^, possibly keeping RS unchanged at lower levels, but decreasing (or increasing) it at higher levels. This modulation in higher levels results from RS itself being better (or worse) explained away, and would depend on expectations, attentional load, etc. Indeed, a recent study^63^ showed an early attention-independent RS effect for an auditory stimulus (around 100 ms), while a later component (around 200 ms) showed RS only when the stimulus was fully attended. Likewise, another study^64^ showed that RS was mainly involved in early and late components of MEG activity, while the predictive context was involved only in medial and late components (after 200 ms). The specific moment and sites where the modulation of RS occurs may indicate the influence of top-down mechanisms in specific stages of processing^63^. It might also explain why the RPE mingles with RS effects in the fMRI literature, due to the poor time resolution of this method.

In summary, the current study shows that an early RPE, close to visual areas, occurs independently of perceptual consciousness of the prime stimulus. Therefore, it excludes the simple account of RPEs as a by-product of conscious strategies. However, the later theta-band RPE, which occurs in medial-frontal sites, was present only when the prime was consciously perceived. While the later might suggest the use of conscious strategies for the build-up of expectations, the former show an automatized and more pervasive influence of predictions on sensory brain regions.

## Methods

In Experiment 1 we tested behavioural RPE for conscious and unconscious repetitions. In Experiment 2, we replicated the behavioural paradigm used in Experiment 1 while recording EEG, allowing us to investigate electrophysiological markers of conscious and unconscious RPE. A third behavioural experiment tested whether an unconscious RPE could be observed even when subjects are never exposed to conscious repetitions. All participants were right handed.

### Participants

#### Experiment 1

33 participants (25 female) aged between 21 and 30 (mean of 25) were tested. Two participants were excluded from the analyses for not completing the full experiment. An additional 10 participants were excluded from the analyses because they were able to discriminate the masked prime above chance level. For the reaction time analysis, 4 participants had less than 20 trials per condition after removing outliers (see Behavioural Analyses below), and where therefore excluded from further analyses. Thus, a total of 21 participants were used for the target sensitivity analyses and 17 for the reaction time analysis.

#### Experiment 2

EEG data was recorded from 28 participants (16 females) aged between 20 and 29 (mean of 23) while they performed the behavioural task. Three participants were excluded due to excessive noise in the EEG signal (2 due to slow oscillations and 1 due to excessive blinks) and another 4 participants for discriminating the prime above chance level (see Behavioural Analyses). Again, 2 participants were excluded for having less than 20 trials per condition. In total, 19 participants were used for the EEG analysis.

Since there were no differences in priming between experiment 1 and experiment 2 for both reaction time [F(1, 37) = 4.9e-4; p = 0.99] and target sensitivity [F(1, 42) = 6.3e-6; p = 0.99], data from experiments 1 and 2 were collapsed across participants for behavioural analyses. As a result, a total of 39 participants (17 from experiment 1 and 22 from experiment 2) were used for the reaction time analysis and 44 participants (21 from experiment 1 and 23 from experiment 2) were used for the target sensitivity analysis.

#### Experiment 3

In this behavioural control experiment, 25 participants (14 females) aged between 21 and 28 (mean of 24) were tested. Two participants didn’t finish the experiment; while another 3 were excluded due to them discriminating the prime above chance level (see Behavioural Analyses). As a result, 20 participants were used for both target sensitivity and reaction time analysis.

In all three experiments sample size was estimated based on previous studies on unconscious repetition priming^36,54^ and repetition suppression^25,65^.

### Ethics statement

All participants provided written informed consent to participate in this study and were paid 10€ to participate in experiment 1 and 3 and 40€ to participate in experiment 2. All methods were carried out in accordance with relevant guidelines and regulations. This protocol was approved by the local ethics committee (Comité de Protection des Personnes, Ile-de-France VI, Paris, France).

### Stimuli

All the stimuli were presented in a dim shielded room, using MATLAB (R2012b) and Psychtoolbox (3.0.10). A BenQ XL2420-B LCD screen was used, with background luminosity of 0.5 (all reported contrasts are in Michelson units). Also, Gamma was applied so that the luminance increased linearly with contrast (which was verified using a Konica Minolta LS-100 luminance meter). Gratings were generated using Gabor patches of 1 cycle per degree, a variance of 4.5 degrees and random phases drawn from a uniform distribution. The prime contrast was adjusted through an adaptive procedure (see Procedure and Design), with an average convergence contrast across participants of 0.0756 +− 0.0629. This contrast was used for the unconscious primes (33 ms duration). For the conscious primes (167 ms duration), 3 extra steps (i.e. 0.3) were added to the contrast obtained from the adaptive procedure in order to ensure that they were consciously perceived. The target gratings had a fixed contrast of 0.47. The prime backward mask consisted of concentric white circles repeated every 0.4 degrees, and the forward mask was created from smoothed Gaussian noise patches (Gaussian kernel of 1 degree). All stimuli were presented in an annulus (diameter 3 to 9 degrees) around the fixation square in the centre of the screen, composed of a small black square (0.25 degrees) inside a white square (0.5 degrees). See Fig. 1A for an example. A photodiode was used to capture the moment when the first frame of the prime and the first frame of the target appeared, and this information was sent to an Arduino UNO and later stored in the computer running Psychtoolbox.

### Procedure and design

Each trial started with a waiting period of 250 ms, and was followed by a sequence of 2 gratings. The first grating lasted either 33 ms (unconscious prime) or 167 ms (conscious prime), while the second grating lasted 250 ms (target). The gratings were always tilted by either −45 degrees (“tilted left”) or +45 degrees (“tilted right”). The two gratings were separated by an ISI of 50 ms where a backward mask was presented. Before the first grating there was also a forward mask that lasted either 167 ms (unconscious prime) or 33 ms (conscious prime), in a way that the duration of the 3 stimuli before the target (i.e. forward mask, prime and backward mask) had a fixed duration of 250 ms (see Fig. 1A).

Participants were instructed to fixate all the time in the fixation square, and answer as fast as possible if the second grating was tilted to the right or to the left, using their left hand and one of the two buttons in the response box. The response box was built in-house using an Arduino UNO and a photodiode, which measured the difference between the frame where the target appeared and the moment where the participant pressed the button. In order to help participants to identify the correct moment to respond, since the first grating could be unconscious, the fixation square inverted contrast when the second grating (target) appeared. After the presentation of the second grating, a blank screen was displayed until response.

After responding to the direction of the target, they were presented with 3 letters G for left (“Gauche”) and 3 letters D for right (“Droite”), equally spaced in a vertical line centred in the fixation point (See Fig. 1A). The letters had 3 different sizes, and participants were instructed to indicate the direction of the first grating using the following criteria: select one of the big letters if they were sure that they saw the direction of the prime, one of the medium if they were not sure and one of the small letters if they were guessing. These Perceptual Awareness Scale (PAS)^66^ is further discussed in the *Behavioural Analyses* section. The cursor appeared upon a random position during each trial. Also, the direction (letters G on top and D in the bottom or vice-versa) was randomly chosen for each trial, to avoid selection bias. They were also instructed to take as long as necessary to select the letter that best represented their impression of the prime. Finally, for experiment 2, with EEG recordings, they were instructed to blink (if necessary) *after* selecting the letter and *before* clicking in the response. After this second response, a blank screen was presented for a fixed interval of 250 ms, and was followed by the next trial.

The experiments involved a 2 × 2 × 2 design with factors of prime visibility (conscious and unconscious), prime-target contingency (repeated and unrepeated) and within block repetition probability also called context (congruent and incongruent).

Participants arrived and received instructions of how to participate in the experiment. After that, they performed 3 training blocks where they were progressively introduced to the stimulus and the tasks. Next, they performed an adaptive block where the prime always lasted 33 ms and the contrast started at 1 Michelson. After each trial, the contrast decreased after a correct discrimination of prime direction (independently of confidence level), and increased after an incorrect discrimination, always by a step of 0.1 in logarithmic scale. The adaptation block stopped after 100 inversions and lasted on average 112 +− 12 trials. The contrast of the prime was then set to the average of the last 20 inversions, and kept constant throughout the experiment (see Stimuli). The probability of repetition for these first 4 blocks (training and adaptive) was drawn from a uniform distribution.

During the last 6 blocks for experiment 1 or 10 blocks for experiment 2 and 3, the probability of prime-target contingency was manipulated, and each block had either: 80% repeated trials (congruent context) or 20% repeated trials (incongruent context). The blocks of each context were always blocked, and the order of presentation was counterbalanced across participants (half the participants started with congruent blocks and finished with incongruent blocks, and the other half the opposite). For experiment 2 and 3, the first block of each context, called transition block, had only 40 trials and was not considered in the analyses. The remaining blocks were composed of 120 trials. All the data presented in this article was collected in these blocks, resulting in a total of 6 blocks (720 trials) for experiment 1 and 8 blocks (960 trials) for experiments 2 and 3, per subject. For experiments 1 and 2, the direction of the gratings (tilted left or tilted right), and the visibility of the prime (conscious or unconscious), was counterbalanced within these blocks, with equal probability for each direction and visibility. For experiment 3 the direction was also counterbalanced, but the prime was *always* unconscious.

### EEG acquisition and preprocessing

During experiment 2, the electroencephalogram was continuously recorded from 64 Ag/AgCl electrodes (Electrical Gegodesic Inc system), with Cz as reference. The impedance for electrodes was kept under 20 KΩ. Data were acquired with a sampling rate of 250 Hz. All the analysis was done using SPM, EEGLAB, Fieldtrip and in-house matlab scripts. Continuous data were high-pass filtered with filedtrip one-pass zero-phase FIR filter, with optimized order and cutoff at 0.2 Hz.

For the ERP analysis, the data was then epoched from −1600 to 1600 ms in relation to target onset, low-pass filtered also with filedtrip one-pass zero-phase FIR filter, optimized order and cutoff at 30 Hz, resized to −100 to 700 ms to avoid border effects, and baseline corrected with respect to the pre-stimulus window. Trials with any electrode passing an absolute threshold of 500 μV were rejected from the analysis. Next, independent component analysis (ICA) was used to unmix the data into 64 components using EEGLAB. Time course and scalp map were visually inspected to identify components associated with eye movements and blinks, and EEG data was projected into electrode space without these components (mean of 4 ± 2 components rejected per participant). After visual inspection, trials containing non-stereotyped artefacts were also removed. On average, 7 ± 7% of trials were rejected per participant.

For the Time-Frequency analysis, the data was epoched from −2500 to 2500 ms in relation to target onset, re-projected to electrode space without the previously identified artifactual components, and also visually inspected for non-stereotyped artefacts. Next, the average from each condition for each participant was subtracted from individual trials before the transformation, so that the transformed activation reflects induced activity, not time-locked to the target (i.e. variable phase). The data was then Wavelet transformed using fieldtrip (width = 3 cycles) from 3 Hz to 8 Hz in logarithmically equal intervals. To reduce artefacts, wavelet values were trimmed at 5 SD (gwidth = 5). All the averages across trials in the time-frequency domain first trimmed the 5% most extreme outliers.

### Behavioural analyses

Since reaction times are known to be lognormal^67^, they were log-transformed before statistical analysis and re-projected into milliseconds for visualization. Trials more than 3 s.t.d. from the mean (less than 2% in all experiments) and trials where participants made a mistake during the main task were rejected from reaction time analysis. Finally, for analysing participants performance while identifying unconscious cues, we first performed an ANOVA on cue performance with the PAS levels as factor, expecting that participants would perform better when reporting the highest level in the PAS scale (meaning “I’m certain I saw the direction of the cue”) vs reporting the lowest level in the PAS scale (meaning “I’m certain I did not see the direction of the cue”). Nevertheless, no significant interaction between PAS level and cue performance was found for experiment 1 and 2 [F(2, 112) = 0.243, p = 0.785] or for experiment 3 [F(2, 38) = 1.184, p = 0.317]. One possible cause of this lack of effect is that we performed a fairly long adaptive procedure and decreased cue contrast at individual level, until cue identification was completely at chance. After convergence, this procedure may have resulted in contrasts that are too low to provide any meaningful PAS scale information, which has been shown to be particularly true for cue durations less or equal to 50 ms^66^. In other words, the selection of PAS levels was performed randomly by participants.

For these reasons, we then collapsed all PAS levels, and calculated one d’ to assess the cue performance per target direction and per context. This procedure removes potential bias where participants responded to the prime direction using the target direction, and hence artificially boosting performance because of the repetition probability manipulation^68^. It is important to notice that another possibility is to average d’ only per target direction. This would avoid a possible confound of different visibility levels per context, but would however require two statistical tests per participant, doubling the amount of data rejected by chance. Moreover, there is no qualitative difference in the main effect when this stricter criterion is used (see Supplementary Fig. S1). We therefore averaged the 4 d’s to obtain one d’ per participant. For each participant, a threshold d’ was calculated by simulating 10000 random sequences drawn from a binomial distribution Bin (N, 0.5), with N being equal the number of trials used for this participant. The value separating the 10% biggest d’s encountered in the surrogated distributions was termed d_t and used as threshold for visibility per subject. All participants with D’ bigger than their d_t were rejected (average d_t 0.4 +− 0.03, roughly equivalent to 58% correct).

### EEG clustering permutation analysis

Statistical significance was assessed through cluster/permutation statistics calculated within participants, allowing us to deal with the potential issue of multiple comparisons in a principled manner. Each cluster was constituted by the samples that consecutively passed a specified threshold (in this case sample p-value of 0.05). Importantly, this threshold doesn’t change the type-1 error, and the method controls for false alarms independently of this value^69^. The cluster statistics was chosen as the sum of the paired t-values of all the samples in the cluster. When testing for interactions, the pairs were formed by the differences between conditions. Then, we compared the cluster statistics of each cluster with the maximum cluster statistics of 3000 random permutations.

For the ERP study, clusters were constructed using the samples that consecutively passed the threshold through time (inside the interval from 0 to 700 ms), while in the time-frequency study the samples were required to pass the threshold consecutively in time and frequency (inside the interval from 0 to 700 ms and from 3 to 8 Hz).

## Electronic supplementary material

Supplementary Information
