# Supplementary material for: Prior Expectation Modulates Repetition Suppression without Perceptual Awareness
Source: Sci Rep. 2018 Mar 22;8:5055. doi: 10.1038/s41598-018-23467-3 (PMC5864919; doi:10.1038/s41598-018-23467-3)
Supplement: Supplementary file 1 — Supplementary Information [file 41598_2018_23467_MOESM1_ESM.pdf]

Prior Expectation Modulates Repetition Suppression without Perceptual Awareness

Supplementary Information

Leonardo Barbosa[1-2] and Sid Kouider[1]

[1] Brain and Consciousness Group (ENS, EHESS, CNRS), École Normale Supérieure - PSL Research University, Paris, France

[2] École Doctorale Cerveau Cognition Comportement, Université Pierre et Marie Curie, Paris, France

Send correspondance to: Leonardo S. Barbosa, leonardo.barbosa@pobox.com

Robustness of the RS effect

In order to verify the robustness of the RS effect in each experiment, we performed further post-hoc analyses in experiments 1 and 2 separately. For experiment 1, this analysis revealed that unconscious priming is significant during the congruent context (36.91ms, $P < 0.05$) and failed to reach significance during the incongruent context (-3.23ms, $P > 0.1$). For experiment 2, unconscious priming was only marginally significant during the congruent context (9.19ms, $P < 0.1$), while again unconscious priming failed to reach significance during the incongruent context (5.29ms, $P > 0.1$). Importantly, experiment 3 had approximately the same number of participants as experiment 1 or experiment 2. However, in experiment 3 post-hoc analyses revealed that unconscious priming was *significant during both contexts*.

One possibility to increase the statistical power in this analysis is to be more lenient with respect to visibility. If we change the visibility threshold and compute the d_t threshold (see *Behavioural Analyses* in the Methods section) to be the value separating the largest 1% d's of the surrogate distribution (instead of 10%, meaning only exclude participants with higher performance), participants are still at chance identifying the cue (mean performance 0.49, $P > 0.1$). However, post-hoc analysis of experiment 2 now reveal that unconscious priming is significant during congruent context (11.39ms, $P < 0.05$) and still failed to reach significance during incongruent context (5.71ms, $P > 0.1$). The same results still hold for experiment 1 (38.78ms, $P < 0.01$ and -4.84ms, $P > 0.1$, respectively). Importantly, during experiment 3, unconscious priming is still significant in both contexts (11.57ms, $P < 0.05$ and 9.47ms, $P < 0.05$, respectively). As argued in the discussion of the main text, if lack of statistical power of the RS effect was an issue, it is likely that the lack of interaction during experiment 3 would steam from a null result in the RS effect during both conditions, and not the opposite. We argue that our behavioral results are unlikely to steam from the increased statistical power in the merged experiments 1 and 2.

Visibility assessment

As explained in the Methods section, in order to assess prime visibility, we computed the d' for the performance on the prime during each context and target direction (Supplementary Fig. S1). Next, we averaged these values per participant, and compared the average to that extracted from a surrogate distribution. This analysis allows us to remove the influence of target direction over prime performance, and evaluate the actual prime sensitivity[1]. The rational is that If participants systematically respond according to the direction of the target (or possibly to the opposed direction), the d' will be zero when the target has the same direction as the signal (since hits will be 1 but false alarms will also be 1) and zero when the target has the direction opposite to the signal (both hits and false alarms will be zero).

Another possibility is to exclude participants that are above chance during any one of the two contexts, averaging only over target directions. This would exclude the possibility of confounds, where participants are aware during one context but unaware during another. However, since two statistical tests will be performed, it is expected that the number of participants rejected by chance (i.e. participants that are indeed unaware of the prime direction) will double. Indeed, this stricter rejection criteria reject another 10 participants. Nevertheless, even after a 30% decrease in the number of participants, not only the main interaction is still marginally significant [$F(1, 28) = 4.04$; $P < 0.1$], but also post-hoc analyses

confirm that unconscious priming is still significant during congruent context (10.59 ms, $P < 0.05$) while unconscious priming still failed to reach significance during the incongruent context (-0.54 ms, $P > 0.1$). It is important to notice that as previously discussed, being so strict about visibility can seriously affect statistical power. Moreover, the results above show that the asymmetry in prime visibility across contexts is unlikely to drive changes the main effect. For these reasons, we maintain the rejection criterion used in the main text (i.e. the average of d-prime across both contexts and target directions).

Supplementary Figure S1

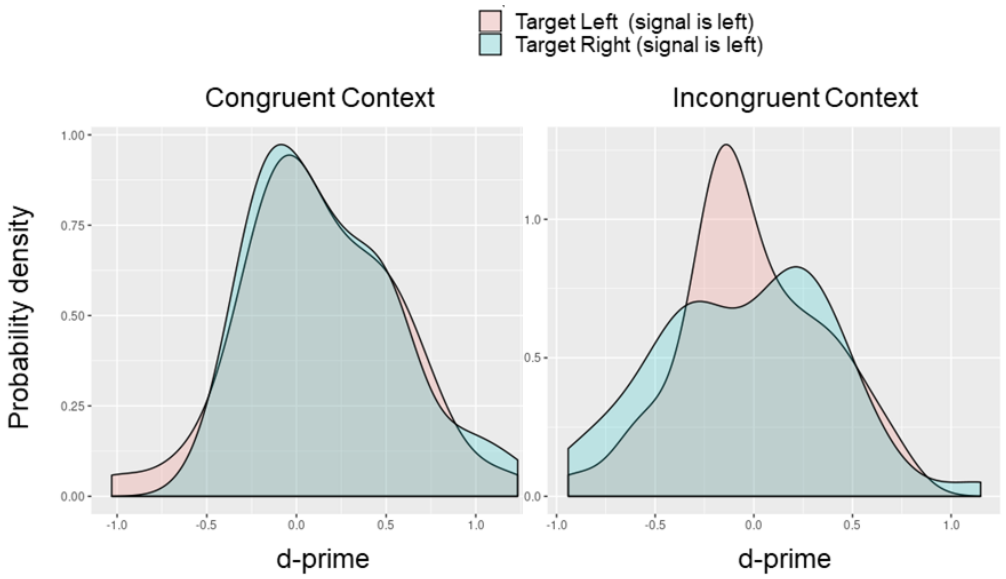

Figure Legends

Supplementary Figure 1. Visibility assessment. Probability density for the distribution of d-primes across participants per context and target direction.

References

1. Macmillan, N. A. & Creelman, C. D. *Detection theory: A user's guide*. (Psychology press, 2004).
